# Effects of community-acquired pneumonia on biventricular cardiac functions in children

**DOI:** 10.1186/s13052-025-01965-1

**Published:** 2025-04-17

**Authors:** Rehab Elmeazawy, Esraa Abd Al-Fattah Sorour, Al Shimaa Badreldeen

**Affiliations:** Department of Pediatrics, Faculty of Medicine, Tanta University, Tanta, Egypt

**Keywords:** Children, Pneumonia, Speckle tracking echocardiography, Tissue doppler imaging

## Abstract

**Background:**

This study aimed to evaluate the effects of community-acquired pneumonia (CAP) on cardiac function in children and compare the effectiveness of tissue Doppler imaging (TDI) and two-dimensional speckle tracking echocardiography (2D-STE) with conventional echocardiography in the early detection of biventricular cardiac dysfunction in children with CAP.

**Methods:**

The study included 50 hospitalized children diagnosed with CAP and 50 matched healthy controls. All patients underwent cardiac evaluation including conventional echocardiography, TDI, and 2D-STE.

**Results:**

Fifty children with CAP with a mean age of 5.02 ± 2.46 years participated in the study. Thirty-two were male (64.0%). LV systolic function (S) and RV diastolic function (E′/A′) were significantly lower in the diseased group compared to the control group (*p* < 0.001). The myocardial performance index (MPI) of both ventricles was significantly higher in the diseased group compared to the control group (*p* < 0.001). In patients with CAP, the mean value of two-dimensional longitudinal strain (2D LS) was significantly lower than that of the control group (*p* < 0.001). No statistically significant differences in echocardiographic parameters were observed when comparing complicated and non-complicated CAP subgroups.

**Conclusion:**

TDI and 2D-STE demonstrated the ability to detect early biventricular dysfunction in pediatric patients diagnosed with CAP.

## Background

Pneumonia is a leading cause of morbidity and mortality in children under five years of age. The incidence of community-acquired pneumonia (CAP) among children aged less than 5 years in developing countries reached 0.29 per child per year, with a mortality rate of 1.3–2.6% [1].

Every year, an estimated 1.1 to 1.4 million children die from pneumonia, accounting for 17 to 19% of all pediatric mortality. Most of these deaths occur in underdeveloped countries. In Egypt, approximately 6% of children experience at least one episode of pneumonia during their first two years of life [2].

Pneumonia can trigger a systemic inflammatory response during acute infection. Dysregulated local and systemic inflammation persists even after the successful eradication of the infecting pathogen in the lungs, leading to longer-term deleterious effects on cardiovascular structures [3].

The increased concentrations of inflammation mediators during the acute phase of pneumonia such as tumor necrosis factor-α, interleukin-6, endothelin-1, B-type natriuretic peptide, and atrial natriuretic peptide have direct negative effects on cardiomyocytes by suppressing their function and inducing cell death leading to left ventricular dysfunction [4].

Some research groups have identified that S. pneumoniae, the most frequent isolated pathogen in patients with CAP, can generate a direct cardiotoxic effect by invading the myocardium and creating microlesions, as well as by the direct cytotoxic effect of virulent factors such as pneumolysin and cell wall [5]. Pneumolysin disrupts calcium homeostasis in cardiomyocytes, which can lead to abnormal ion flow (e.g., potassium, sodium, magnesium), resulting in arrhythmias, cardiac arrest, and heart failure [6, 7].

Mycoplasma pneumoniae, an etiology of atypical pneumonia, has the capability of reaching the heart and triggering pericarditis as well as myopericarditis in adults and children with pneumonia. In addition, macrolides and fluoroquinolones used in the treatment of mycoplasma pneumoniae can also induce QT prolongation and, rarely, polymorphic ventricular tachyarrhythmia [8]. However, most subjects experiencing cardiac arrhythmias from macrolides have coexisting risk factors and the incidence of arrhythmia in the absence of coexisting risk factors is very low [9].

Hypoxia caused by pneumonia increases the risk of cardiovascular complications by affecting cardiac and vascular function. This can lead to pulmonary vasoconstriction, resulting in pulmonary hypertension and right ventricular dysfunction. Research involving young and middle-aged patients has demonstrated that pneumonia can increase pulmonary artery pressures in proportion to the severity of ventilatory restriction and hypoxemia [10].

CAP leads to increased levels of serum vasopressin with impairment of renal water excretion which causes the syndrome of inappropriate antidiuretic hormone secretion leading to hyponatremia and electrolyte imbalance that induce cardiac arrhythmias [11]. Besides, pneumonia itself could cause acute renal injury and worsening of electrolyte imbalance and volume overload that may predispose to heart failure [12].

Acute pneumonia can cause a wide range of acute changes in the surface electrocardiogram, leading to cardiac arrhythmias that result from a direct effect of pneumonia on the cardiac conduction system, concomitant myocardial disorders such as ischemia or myocarditis, or pericardial involvement by pneumonia-producing organisms [13]. Atrial tachyarrhythmias, particularly atrial fibrillation, are a frequent early complication of patients admitted with CAP, which may be attributed to the increased risk of thromboembolic complications in CAP [14].

Anti-infective agents, such as antibacterial and antiviral medications, can cause cardiotoxicity, which may range from arrhythmias to myocardial damage and even heart failure. The severity of these effects on the heart depends on the level of exposure and varies among patients. Factors such as gender, age, and genetic background can influence how individuals respond to these medications [15].

However, antibiotics target and eliminate the bacteria causing the infection, reducing the overall inflammatory response in the body. This helps prevent the spread of infection to the heart and other organs [16]. In addition, treating the infection improves lung function and oxygenation, reducing the strain on the heart and preventing complications like heart failure [17].

The purpose of this study was to evaluate the effect of pediatric community-acquired pneumonia on cardiac function and to compare tissue Doppler imaging (TDI) and two-dimensional speckle tracking echocardiography (2D-STE), versus conventional echocardiography in early detection of biventricular cardiac dysfunction in pediatric pneumonia.

## Methods

This case-control study included 50 hospitalized children with a diagnosis of CAP admitted to the pediatric department from February 2023 to January 2024. In addition, 50 matched healthy controls were included who showed no significant differences in age and gender distribution compared to the patient group.

This study was performed in line with the principles of the Declaration of Helsinki and its later amendments. The research ethics committee of the Faculty of Medicine, Tanta University has approved this research under the number 36264PR29/1/23. Written informed consent was obtained from the patients’ guardians.

Based on a previous study by Metawee et al., 2022. To detect the difference in the mean of MPI between groups with a power of 95% and a level of significance of 5% with an effect size of 0.76, a total sample size of 94 participants in each group. To compensate for a possible dropout 10% will be added, therefore the total sample size in each group will be 50. The sample size was calculated by G* Power (version 3.1.9.4).

All children between the ages of two months and 18 years who met the diagnostic criteria of CAP were included in the study. CAP was defined as the presence of signs and symptoms of pneumonia in a previously healthy child who acquired an infection outside the hospital, as well as radiographic findings of pulmonary consolidation [18]. Complicated pneumonia is characterized as CAP which is further complicated by the presence of para-pneumonic effusion, empyema, necrotizing pneumonia, pneumatoceles, and lung abscess [19].

Children with underlying chronic lung disease, cardiac disease, immunodeficiency disorders, renal insufficiency, liver disease, neurological disease, and malignancy, as well as patients with previous hospitalizations within 1 month, were excluded.

Demographic data such as gender, age, characteristics of the first clinical manifestations, age at diagnosis, antibiotic therapy, length of hospital stay, occurrence of complications, and the need for surgical intervention or insertion of a chest tube were collected. Laboratory tests including CBC, D-dimer, CRP, and pleural fluid analysis and culture were included. A chest X-ray was performed in all patients, while a CT scan of the chest was only performed when complicated CAP is anticipated.

Echocardiographic Examination.

For all cases, echocardiographic examination was performed with real-time three-dimensional 3.5 MHz, S7, and V3 matrix probes on Vivid 7 and Vivid 9 from GE Health Care, Horten, Norway. Digital loops were stored on the hard drive of the echocardiography machine and transferred to a workstation (Echo PAC PC, 113, GE, Horten, Norway) for offline analysis. For conventional echocardiography, TDI, 2D-STE, and 3D-STE, the American Society of Echocardiography guidelines were followed [20].

### Conventional echocardiography

The examination consisted of M-mode, 2D, pulsed, continuous, and color Doppler blood flow velocity measurements of the heart valves. The measurements were taken at the level of the tips of the mitral valve leaflets in the parasternal long-axis view of the left ventricle. Left ventricular dimensions (LVEDD and LVESD), left ventricular fractional shortening (FS), and ejection fraction (EF) were measured, and left ventricular diastolic function was assessed by mitral valve E/A ratio (MV), where E (passive LV filling) and A wave (atrial contraction). The mean pulmonary artery pressure (PAP) was determined based on the Doppler peak signal of tricuspid regurgitation plus right atrial pressure that assumed to be 5 mmHg is for all participants.

### Pulsed Wave- tissue doppler imaging (PW-TDI)

PW-TDI was assessed using the apical four-chamber view. We made sure to increase the frame rate to more than 180 frames /second and to minimize the interrogation angle to the target chamber wall to less than 15°. Tissue Doppler images were acquired from both ventricles at the annulus of the tricuspid valve (TV) and the septal annulus of the mitral valve (MV). The S wave represented the systolic function, while the E′/A′ ratio represented the diastolic function. The myocardial performance index (MPI) assesses both systolic and diastolic ventricular function, MPI = (ICT + IRT)/ET, where ICT is the isovolumetric contraction time, IRT is the isovolumetric relaxation time, and ET is the ejection time [21].

### Speckle tracking echocardiography (STE)

2D STE images were acquired at a frame rate of > 40 frames/cycle during three cardiac cycles. An image of the apical chamber was used to measure global LV longitudinal strain. The endocardial border was automatically monitored using the software after changing the 2D planes. However, if more detailed tracking is required, this can be changed manually by the operator.

Intra-observer variability was assessed in 20 randomly selected patients by repeated analysis on the same film loop.

The operator can correct the shape of the region of interest by using attractor points to pull the nearby region of interest border towards the user’s desired location from the tracking results. Negative values resulted from thinning or shortening.

### Statistical analysis

Data were loaded into a Microsoft Excel spreadsheet and examined using version 20 of SPSS software. Percentages and numbers were used to describe the data. The normality of the distribution was checked using the Kolmogorov-Smirnov test. Normally distributed quantitative data were described as means ± standard deviation (SD) and compared using Student’s t test, while abnormally distributed quantitative variables were described as median and interquartile range (IQR) and compared using the Mann-Whitney test. Chi-square was used to compare qualitative data in both groups. ROC curve analysis was used to detect CAP. Statistical significance was determined at a P value of < 0.05.

## Results

### Clinical and laboratory data

Fifty children with CAP with a mean age of 5.02 ± 2.46 years participated in the study. Thirty-two were male (64.0%). Compared to the control group (50 healthy children), the diseased children had a significantly higher respiratory rate, WBC count, neutrophil, monocyte, lymphocyte, and platelet count. NLR, MLR, PLR, blood urea, and serum creatinine were also significantly higher in the diseased group (*p* < 0.001). While the hemoglobin level, O2 saturation, and serum albumin were significantly lower in the diseased group (*p* < 0.001) (Table 1).

**Table 1:** Demographic and laboratory data of the studied groups

	Patients (*n* = 50)	Control (*n* = 50)	*P* value
Sex Male Female	32 (64.0%) 18 (36.0%)	26 (52.0%) 24 (48.0%)	0.224
Age (year)	5.02 ± 2.46	4.46 ± 2.45	0.254
Respiratory rate (breath/min)	48.72 ± 10.0	25.56 ± 4.51	< 0.001*
O2 Saturation %	94.04 ± 2.58	96.62 ± 1.72	< 0.001*
Hemoglobin (gm/dl)	10.25 ± 1.31	12.33 ± 1.01	< 0.001*
WBCs (10^3^/µL)	20.18 ± 9.77	7.17 ± 1.16	< 0.001*
Neutrophils (10^3^/µL)	15.21 ± 8.71	3.65 ± 0.69	< 0.001*
Lymphocytes (10^3^/µL)	3.86 ± 1.89	2.61 ± 1.04	< 0.001*
Monocytes (10^3^/µL)	0.77 ± 0.35	0.30 ± 0.13	< 0.001*
Platelet (10^3^/µL)	475.76 ± 200.66	259.72 ± 69.52	< 0.001*
NLR	4.46 ± 2.46	1.68 ± 0.81	< 0.001*
MLR	0.22 ± 0.09	0.13 ± 0.08	< 0.001*
PLR	156.71 ± 105.31	121.55 ± 63.37	0.046*
AST (IU/L)	27.32 ± 11.30	26.08 ± 4.15	0.467
ALT (IU/L)	23.47 ± 6.92	22.16 ± 3.15	0.225
Blood urea (mg/dl)	28.54 ± 11.00	17.52 ± 3.44	< 0.001*
Serum creatinine (mg/dl)	0.67 ± 0.17	0.42 ± 0.05	< 0.001*
Serum albumin (gm/dl)	3.50 ± 0.31	4.28 ± 0.45	< 0.001*

*P < 0.05 significant, WBCs: white blood cells, NLR: neutrophil/lymphocyte ratio, MLR: monocyte/lymphocyte ratio, PLR: platelet/lymphocyte ratio

Patients were divided into non-complicated (22 children, 14 male) and complicated (28 children, 18 male) groups. When comparing the two groups in terms of age and clinical data, including fever, grunting, chest pain, use of accessory muscles, respiratory rate, and cough, no significant differences were observed. However, gastrointestinal manifestations were notably more prevalent in the complicated subgroup (Table 2). A significant difference was observed between complicated and non-complicated CAP in terms of the location of the affected lung lobe, with the right lower lobe being more affected in the complicated group (42.9%) than in the non-complicated group (36.4%).

**Table 2:** Demographic and clinical data of patients with CAP

	Non-complicated CAP (*n* = 22)	Complicated CAP (*n* = 28)	*P* value
Sex Male Female	14 (63.6%) 8 (36.4%)	18 (64.3%) 10 (35.7%)	0.962
Clinical manifestations Fever Grunting Chest pain Accessory muscle use Cough GIT manifestations	20 (90.9%) 10 (45.5%) 2 (9.1%) 16 (72.7%) 8 (36.4%) 4 (18.2%)	26 (92.9%) 14 (50.0%) 8 (28.6%) 22 (78.6%) 18 (64.3%) 16 (57.1%)	0.801 0.749 0.087 0.631 0.05 0.005*
Ineffective antibiotics	4 (18.2%)	10 (35.7%)	0.171
Lung lobe involved Right upper lobe Right upper &middle lobes Right middle &lower lobes Right lower lobe Whole right side Left lower lobe Whole left side Bilateral lobar	2 (9.1%) 2 (9.1%) 0 (0.0%) 8 (36.4%) 4 (18.2%) 4 (18.2%) 0 (0.0%) 2 (9.1%)	0 (0.0%) 0 (0.0%) 3 (10.7%) 12 (42.9%) 8 (28.6%) 1 (3.6%) 4 (14.3%) 0 (0.0%)	0.021*
Surgical intervention Chest tube Chest tube + Decortication Decortication		10 (35.7%) 4 (14.3%) 4 (14.3%)	
Combined used antibiotics Cephalosporin-Ampicillin sulbactam Cephalosporin-Vancomycin Cephalosporin-Linezolid Carbapenem-Linezolid Levofloxacin-Linezolid Cephalosporin-Clindamycin	16 (72.7%) 2 (9.1%) 4 (18.2%) 0 (0.0%) 0 (0.0%) 0 (0.0%)	4 (14.3%) 8 (28.6%) 9 (32.1%) 3 (10.7%) 2 (7.1%) 2 (7.1%)	0.002*
Macrolides	8 (36.4)	8 (28.6%)	0.558

*P < 0.05 significant, CAP: community-acquired pneumonia, GIT: gastrointestinal

Ten cases (35.7%) of the complicated group required the insertion of a chest tube, four cases (14.3%) required the insertion of a chest tube and decortication, and four cases (14.3%) required decortication only (Table 2).

In non-complicated CAP, combined cephalosporin and ampicillin-sulbactam were the most commonly used antibiotics. In cases of complicated CAP, combined cephalosporin with vancomycin or linezolid was frequently utilized (*P* = 0.002*). There were no differences in the use of macrolides between both groups, and neither group of patients experienced any complications during their treatment.

Compared with non-complicated CAP, the duration of hospital stays, and duration of fever were statistically significantly higher in the complicated group (*P* < 0.001). When comparing the complicated and non-complicated subgroups based on laboratory data, only the platelet count was significantly higher in the complicated group. All other parameters, including hemoglobin, WBCs, neutrophils, lymphocytes, monocyte count, NLR, MLR, PLR, CRP, and D-dimer levels, showed no significant differences between the two groups (see Table 3).

**Table 3:** Laboratory data of the patients with CAP

	Non-complicated CAP (*n* = 22)	Complicated CAP (*n* = 28)	*P* value
Age (year)	4.55 ± 2.19	5.16 ± 2.54	0.472
Duration of hospital stay (day)	8.73 ± 4.84	14.93 ± 5.14	< 0.001*
Duration of fever (day)	2.09 ± 0.68	4.21 ± 1.93	< 0.001*
Respiratory rate (breath/min)	48.45 ± 9.91	48.93 ± 10.25	0.870
O2 Saturation %	93.93 ± 2.908	94.17 ± 2.188	0.738
Hemoglobin (gm/dl)	10.13 ± 1.43	10.34 ± 1.23	0.570
WBCs (10^3^/µL)	18.61 ± 9.43	21.41 ± 10.03	0.319
Neutrophils (10^3^/µL)	13.58 ± 7.36	16.50 ± 9.58	0.243
Lymphocytes (10^3^/µL)	3.62 ± 1.98	4.05 ± 1.83	0.427
Monocytes (10^3^/µL)	0.76 ± 0.37	0.78 ± 0.34	0.881
Platelet (10^3^/µL)	374.55 ± 167.13	555.29 ± 190.90	0.001*
NLR	4.24 ± 1.85	4.64 ± 2.87	0.571
MLR	0.23 ± 0.07	0.021 ± 0.11	0.449
PLR	136.92 ± 96.01	172.62 ± 111.29	0.243
CRP	118.15 ± 87.64	145.99 ± 91.20	0.281
D-dimer	2.61 ± 2.37	2.36 ± 1.05	0.691
AST (IU/L)	25.69 ± 7.875	29.23 ± 14.291	0.273
ALT (IU/L)	22.73 ± 4.677	24.35 ± 8.912	0.415
Blood urea (mg/dl)	29.00 ± 11.835	28.00 ± 10.171	0.752
Serum creatinine (mg/dl)	0.69 ± 0.201	0.64 ± 0.133	0.250
Serum albumin (gm/dl)	3.55 ± 0.366	3.45 ± 0.229	0.261

*P < 0.05 significant, CAP: community-acquired pneumonia, WBCs: white blood cells, NLR: neutrophil/lymphocyte ratio, MLR: monocyte/lymphocyte ratio, PLR: platelet/lymphocyte ratio, CRP: C-reactive protein

### Echocardiographic examination results

**Fig. 1:**
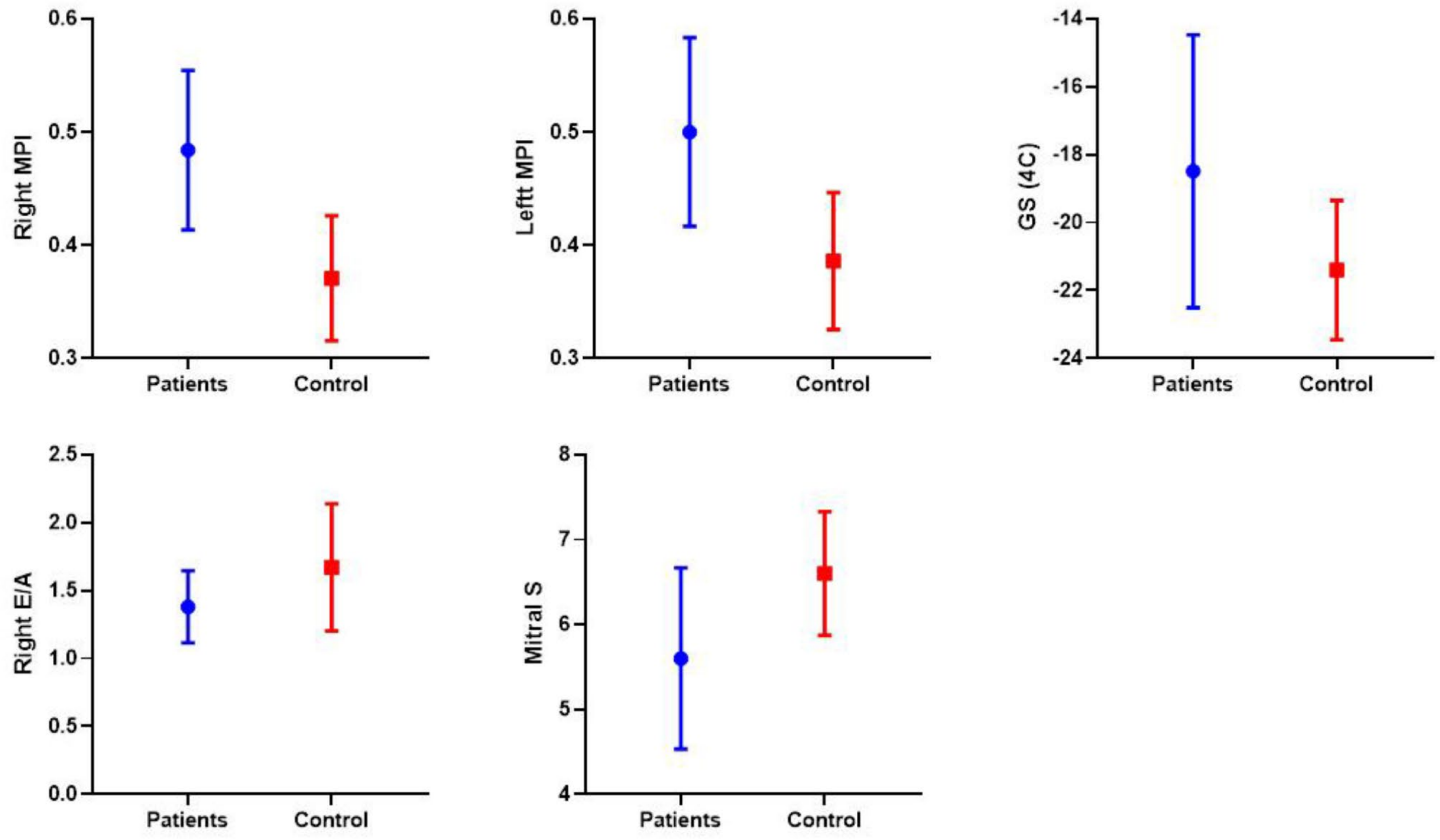
Comparison between patients and controls in terms of TDI significant parameters; Right MPI, Left MPI, Right E/A, Mitral S, and 2D-STE; GS

No significant differences were found in FS measured in M ​​mode, LV diastolic function (E’/A’), and RV systolic function (S) measured via TDI in children with CAP compared to the control group. However, LV systolic function (S) and RV diastolic function (E’/A’) were significantly lower in the diseased group (Table 4; Fig. 1).

**Table 4:** Echocardiographic data of the studied groups

	Patients (*n* = 50)	Control (*n* = 50)	*P* value
Conventional echocardiography FS%	40.09 ± 3.91	39.04 ± 3.16	0.144
Tissue Doppler imaging Mitral S (cm/s) LV E’/A’ LV MPI Tricuspid S (cm/s) RV E’/A’ RV MPI	5.60 ± 1.07 1.44 ± 0.24 0.50 ± 0.08 7.44 ± 1.98 1.37 ± 0.27 0.48 ± 0.09	7.26 ± 1.03 1.49 ± 0.22 0.39 ± 0.05 7.84 ± 1.16 1.66 ± 0.38 0.36 ± 0.05	< 0.001* 0.264 < 0.001* 0.217 < 0.001* < 0.001*
**2D-STE** GS (4 C)	-18.49 ± 4.01	-21.88 ± 2.31	< 0.001*

*P < 0.05 significant, FS: fraction shortening, Mitral S: Systolic function at mitral valve, LV E’/A’: left ventricle Early/Late diastolic velocities, LV MPI: left ventricle myocardial performance index, Tricuspid S: Systolic function at tricuspid valve, RV E’/A’: right ventricle Early/Late diastolic velocities, RV MPI: right ventricle myocardial performance index, 3D-STE: two-dimensional speckle tracking echocardiography, GS (4 C): global strain of 4 chambers

**Fig. 2:**
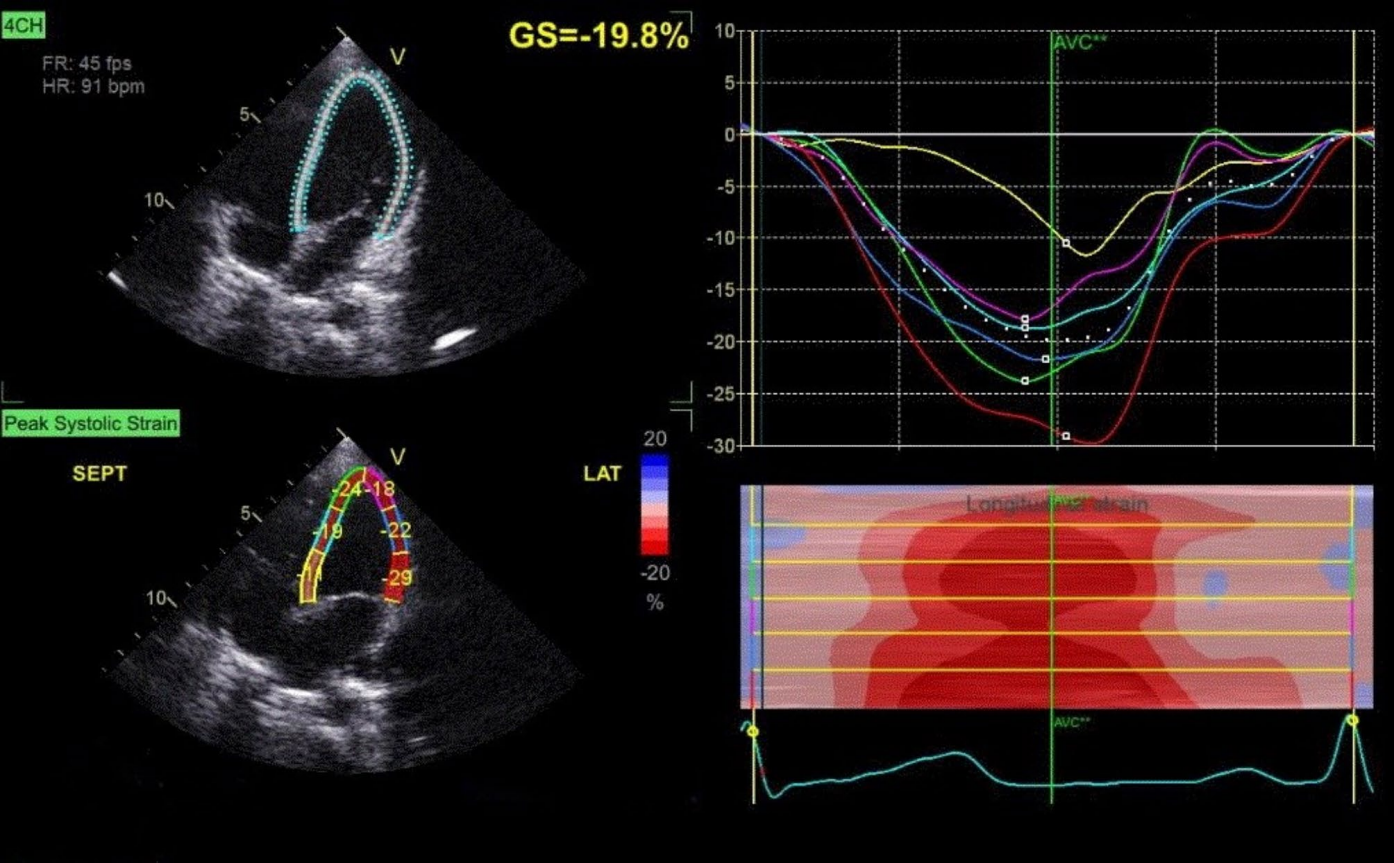
2D-STE of typical 4 chambers view of a patient with CAP showing affected GS of the left ventricle − 15.8%

**Fig. 3:**
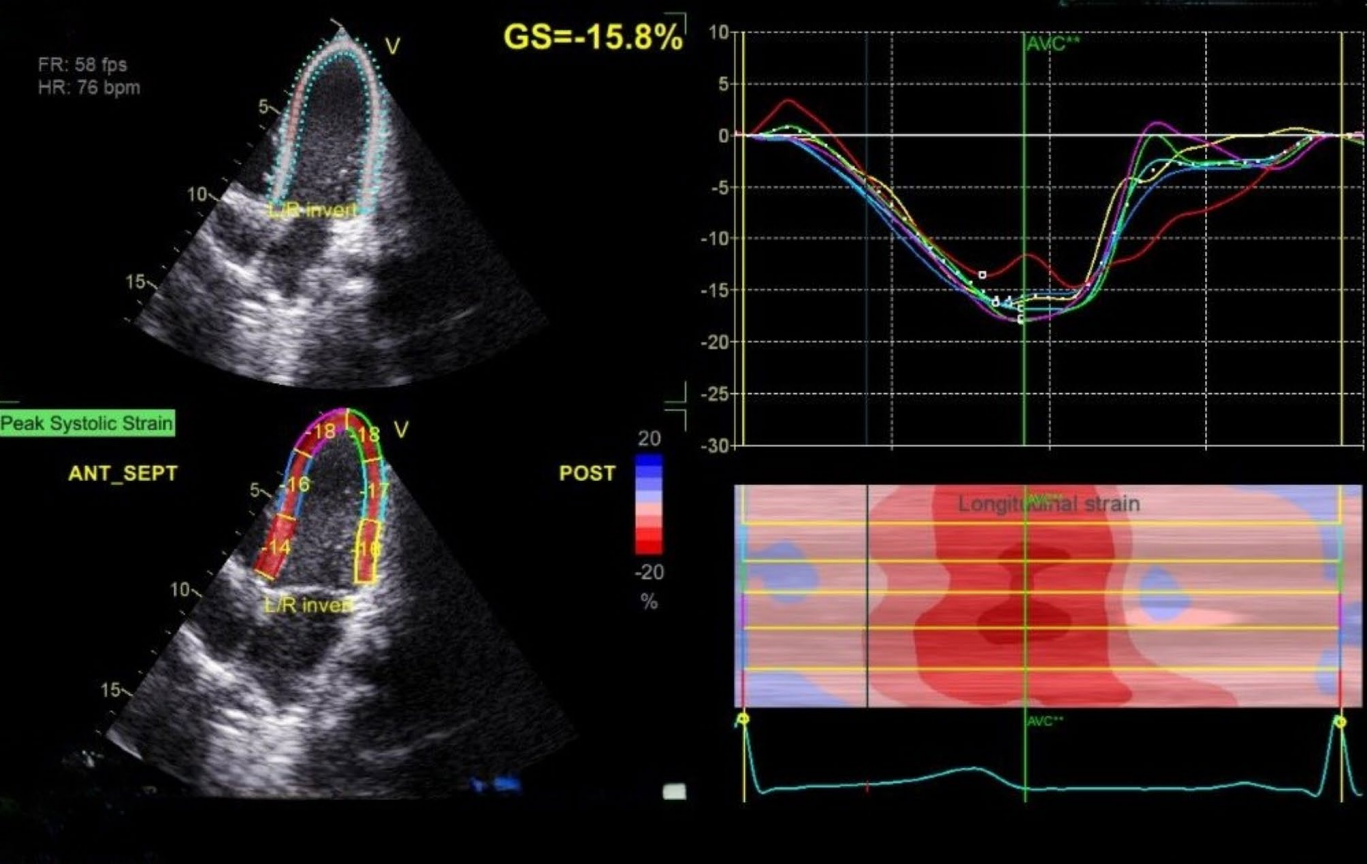
2D-STE of typical 4 chambers view of a healthy child as a control showing affected GS of the left ventricle − 19.8%

The MPI of both ventricles was significantly higher in the diseased group compared to the control group (*p* < 0.001). Compared to the control group, the mean value of two-dimensional longitudinal strain (2D LS) was significantly lower in patients with pneumonia (*p* < 0.001) (Table 4; Figs. 2 and 3).

A comparison between the complicated and non-complicated CAP subgroups revealed no statistically significant differences in ECHO parameters (Table 5).

**Table 5:** Echocardiographic data of the patients with CAP

	Non-complicated CAP (*n* = 22)	Complicated CAP (*n* = 28)	*P* value
Conventional echocardiography FS%	39.49 ± 4.36	40.26 ± 3.82	0.572
Tissue Doppler imaging Mitral S (cm/s) LV E’/A’ LV MPI Tricuspid S (cm/s) RV E’/A’ RV MPI	5.82 ± 1.33 1.50 ± 0.24 0.47 ± 0.09 7.81 ± 2.14 1.46 ± 0.34 0.48 ± 0.08	5.54 ± 0.99 1.42 ± 0.23 0.51 ± 0.08 7.33 ± 1.95 1.35 ± 0.25 1.35 ± 0.25	0.449 0.304 0.154 0.479 0.213 0.879
**2D-STE** GS (4 C)	-18.08 ± 2.31	-18.60 ± 4.39	0.708

*P < 0.05 significant, CAP: community-acquired pneumonia, FS: fraction shortening, Mitral S: Systolic function at mitral valve, LV E’/A’: left ventricle Early/Late diastolic velocities, LV MPI: left ventricle myocardial performance index, Tricuspid S: Systolic function at tricuspid valve, RV E’/A’: right ventricle Early/Late diastolic velocities, RV MPI: right ventricle myocardial performance index, 3D-STE: two-dimensional speckle tracking echocardiography, GS (4 C): global strain of 4 chambers

## Discussion

To the best of our knowledge, there are only a limited number of studies focusing on echocardiographic changes associated with CAP in children. In our research, we assessed cardiac function in children diagnosed with CAP using various echocardiographic modalities, including conventional echocardiography, TDI, and 2D-STE.

LV-related findings suggested significant deterioration in LV systolic function, as evidenced by a decrease in S’ observed by TDI and a decrease in global longitudinal strain (GLS) assessed by 2D-STE. In contrast, no significant differences were found in FS% measured in M ​​mode or LV diastolic function assessed by E’/A’ ratio in children with CAP.

The differences observed in our systolic function results assessed in M-mode as opposed to TDI and 2D-STE may be attributed to the limited sensitivity and specificity of parameters derived from M-mode as well as the advantages of alternative echocardiographic techniques. LV (GLS) facilitates early detection of myocardial dysfunction before significant changes in fractional shortening (FS) or ejection fraction (EF) occur [22].

Our results are consistent with those of Metawee et al., who reported significantly lower S’ in children diagnosed with pneumonia, suggesting the presence of LV systolic dysfunction in these patients [23]. Similar to our results, they observed no significant difference in diastolic function between cases and controls when assessed by TDI. However, their analysis using conventional pulsed Doppler revealed a notable decrease in both the E wave and the E/A ratio in pneumonia cases compared to the control group.

Orabi et al. conducted an assessment of cardiovascular dysfunction in pediatric patients diagnosed with pneumonia [24]. Their study used conventional echocardiography, TDI, and 2D-STE techniques. The results suggested the presence of LV systolic and diastolic dysfunction and LV hypertrophy. Consistent with our results, they observed a reduction in LV GLS. In addition, they found significant impairments in several echocardiographic parameters in their patients, including LV E/E`, LV end-diastolic volume (EDV), aortic strain, and LV interventricular septal thickness during diastole (IVSd).

In contrast to our research, Atta et al. identified notable diastolic dysfunction within the affected group, as evidenced by the septal and lateral E/e’ ratio [25]. However, there was no significant difference in systolic function. Furthermore, Kalra et al. showed that diastolic dysfunction associated with pneumonia occurs before systolic impairment [26].

Conversely, alternative studies suggested that pediatric patients with pneumonia or lower respiratory tract infections had normal LV dimensions and functions in their findings [27–29].

Regarding RV functions, in our research, we noticed a significant RV diastolic dysfunction measured by E’/A’ and MPI, while there was no significant difference in RV systolic function (S’) measured by TDI, compared to the control group. This finding is in contrast to that of Metawee et al. conducted a study that reported a significant decline in RV systolic function assessed using conventional echocardiography by measuring tricuspid annular plane systolic excursion (TAPSE) [23].

Various studies have demonstrated an increase in RV systolic pressure in children diagnosed with pneumonia [28, 29]. Furthermore, Shehan et al. reported that RV heart failure secondary to pulmonary hypertension occurred in 26% of 47 children with pneumonia [30].

According to our results, the MPI for both ventricles was significantly increased in the diseased group compared to the control group. The MPI serves as a measure of systolic and diastolic function and acts as a sensitive marker for symptomatic heart failure. It reflects the severity of ventricular dysfunction and has been established as an independent prognostic factor for mortality [31]. This aligns with the findings of Nimdet K et al., who observed in their research on pediatric pneumonia that there was a notable deterioration in MPI of both the right and left ventricles at the time of admission, indicating improvement showed during the follow-up period [32]. Additionally, Kaya et al. found that the MPI was significantly higher in patients with COVID-19 pneumonia than in controls [33].

In our research, an analysis comparing the subgroups of complicated and non-complicated CAP revealed that there were no statistically significant differences in echocardiographic parameters. This finding is consistent with the conclusions of Metawee et al., who examined echocardiographic parameters in discharged patients compared with deceased patients. They found that pulsed and TDI along with relevant echocardiographic parameters do not serve as predictors of pneumonia outcome [23].

In contrast, Atta et al. discovered a strong association between hypoxia and the severity of diastolic dysfunction [25]. In addition, echocardiographic parameters can serve as predictive indicators of the outcome of pneumonia, as evidenced by a significant decrease in diastolic function in more complicated cases. Similarly, Orabi et al. identified an association between worsening echocardiographic parameters and the severity of pneumonia [24].

The presence of local pulmonary complications is one of the causes of the failure of childhood CAP treatment. So, in our study, we added anti-staphylococcus antibiotics to fasten the duration of recovery which is in agreement with the previous literature which confirmed that combination antibiotic therapy appeared to be associated with lower mortality among severe cases of CAP [34–37].

In children aged 5 years and older, in addition to Streptococcus pneumoniae, another significant bacterial cause of pneumonia is Mycoplasma pneumoniae. Therefore, in this study, we included macrolides in the treatment for both groups of children with CAP. None of the patients in either group experienced any complications during their treatment with macrolides. This aligns with previous research recommending the addition of macrolides to the treatment of CAP due to their anti-inflammatory and immunomodulatory activities, in addition to their antibiotic effects [38, 39].

### Limitations of the study

This study has several limitations. First, it is a single-center case-control study with a relatively small sample size. Second, we did not repeat echocardiography to assess cardiac function or to determine the duration required for improvement. Finally, we did not evaluate other cardiac biomarkers, nor did we assess additional hemodynamic parameters like electrolyte levels.

We recommend incorporating cardiac biomarkers in future research to identify early myocardial damage before functional deterioration. These biomarkers may include N-terminal pro-brain (B-type) natriuretic peptide (NT-proBNP), cardiac troponins (cTns), cTnI and cTnT, Midregional-Proadrenomedullin, Adrenomedullin (ADM), and Endothelin-1 (ET-1).

## Conclusion

Children with CAP may experience early and subclinical biventricular impairment, which is detected by new echocardiographic modalities based on TDI and 2D-STE. In contrast, the extent of cardiac dysfunction does not correlate with the severity of CAP in pediatric patients.

## Data Availability

The datasets used and/or analyzed during the current study are available from the corresponding author upon reasonable request.
